# Anxiety risk SNPs on chromosome 2 modulate arousal in children in a fear generalization paradigm

**DOI:** 10.1007/s00787-019-01458-7

**Published:** 2019-12-21

**Authors:** Julia Reinhard, Carsten Drepper, Heike Weber, Miriam A. Schiele, Katharina Kneer, Anna Mittermeier, Lillien Frey, Andreas Reif, Paul Pauli, Katharina Domschke, Jürgen Deckert, Marcel Romanos

**Affiliations:** 1 Department of Child and Adolescent Psychiatry, Psychosomatics and Psychotherapy, Center of Mental Health, University Hospital of Würzburg, Würzburg, Germany; 2 Department of Psychiatry, Psychosomatics and Psychotherapy, Center of Mental Health, University Hospital of Würzburg, Würzburg, Germany; 3 Department of Psychiatry, Psychosomatic Medicine and Psychotherapy, University Hospital of Frankfurt, Frankfurt, Germany; 4 Department of Psychiatry and Psychotherapy, Medical Center, Faculty of Medicine, University of Freiburg, University of Freiburg, Freiburg, Germany; 5 Department of Psychology (Biological Psychology, Clinical Psychology and Psychotherapy), University of Würzburg, Würzburg, Germany

**Keywords:** Anxiety disorders, Childhood and adolescence, Anxious personality traits, Fear conditioning and generalization, Arousal, Anxiety risk genes

## Abstract

Alterations in fear learning/generalization are considered to be relevant mechanisms engendering the development of anxiety disorders being the most prevalent mental disorders. Although anxiety disorders almost exclusively have their first onset in childhood and adolescence, etiological research focuses on adult individuals. In this study, we evaluated findings of a recent meta-analysis of genome-wide association studies in adult anxiety disorders with significant associations of four single nucleotide polymorphisms (SNPs) in a large cohort of 347 healthy children (8–12 years) characterized for dimensional anxiety. We investigated the modulation of anxiety parameters by these SNPs in a discriminative fear conditioning and generalization paradigm in the to-date largest sample of children. Results extended findings of the meta-analysis showing a genomic locus on 2p21 to modulate anxious personality traits and arousal ratings. These SNPs might, thus, serve as susceptibility markers for a shared risk across pathological anxiety, presumably mediated by alterations in arousal.

**Electronic supplementary material:**

The online version of this article (10.1007/s00787-019-01458-7) contains supplementary material, which is available to authorized users.

## Introduction

Anxiety disorders (AD) represent the most prevalent mental disorders [1] and are typically characterized by an early onset in childhood [2]. AD often persist from childhood into adulthood [3] and are the precursor to a range of other psychiatric disorders, e.g., depression [4]. Since pathological anxiety has long-term negative consequences for child development [5], advancing our understanding of the pathogenic, as well as risk factors and mechanisms of AD, has substantial societal impact by defining concrete points of intervention, and thus ultimately improving treatment efficiency, as well as in aiding preventative and treatment efforts.

In the development of manifest AD many factors play important roles, among others especially personality, environmental, and genetic factors. Although there are different diagnostic definitions relative to their clinical presentation, AD as a group is believed to have a common underlying diathesis relating to an abnormal threat-response regulation [6]. Unlike categorical definitions, which could be afflicted, e.g., with threshold problems, AD can also be considered on a dimensional continuum with respect to symptom intensity, severity, and frequency of occurrence of symptoms and assessed self-report questionnaires symptoms [7, 8].

The National Institute of Mental Health Research Domain Criteria (RDoC) [9] exemplified a change in the way of classifying psychopathology by cutting across traditional nosological divisions, rather than cluster signs and symptoms. Dimensional constructs for studying psychopathology according to this RDoC initiative are, e.g., negative/positive valence systems and arousal/regulatory systems. Autonomic arousal, for instance, is positively correlated with several AD, but also with symptoms of depression [10].

In the pathogenesis of anxiety disorders and threat-response regulation, fear conditioning is a central learning mechanism. Differential fear conditioning (FC) refers to learning that a conditioned stimulus (CS +) predicts an aversive event [the unconditioned stimulus (UCS)], while another stimulus (CS −) is never followed by the UCS and predicts safety. In fear generalization, the conditioned fear responses extend to stimuli (generalization stimuli, GSs) more or less similar to the CS + , but never associated with the UCS. The generalization gradient usually diminishes as a function of reduced similarity between GSs and CS + [11].

There are multiple ways of measuring conditioned fear responses, e.g., self-report ratings and psycho-physiological responses (e.g., skin conductance response). Self-report ratings of arousal level are commonly used for measuring emotional states in humans representing the fear intention. Skin conductance response (SCR) reflects changes in sweat gland activity that alters the electrical conductivity of the skin. As such, it is thus associated with the sympathetic nervous system and covaries with the arousal level.

Disturbances in fear learning are associated with AD, since increased fear responses in underage participants with AD were found both toward CS + as well as CS- [12, 13], indicating impaired safety signal learning in anxious children. Furthermore, abnormalities in fear generalization became the foci of research, since, e.g., generalized anxiety disorder [14] and panic disorder [15] in adults were found to be characterized by greater fear generalization. This endophenotypic approach offers the opportunity to study the psychopathological development of AD as a group on a multilevel approach from subclinical levels with a focus on abnormal threat regulation, as well as dimensional constructs of a negative/positive valence system and arousal/regulatory system. AD [16] and fear conditioning [17] have shown heritability estimates ranging between 30 and 45%. However, although many candidate gene polymorphisms have been investigated in animals and in humans relating to fear learning and AD [18–20], the genetic architecture underlying human maladaptive overgeneralization has been poorly investigated. Additionally, it remains largely unknown which susceptibility genes play a role in subjective and objective arousal during fear learning and its generalization.

Most genome-wide association studies (GWAS) have been performed for specific disorders rather than clusters of disorders with continuous liability distribution, even if there are shared genetic risk factors between disorders and substantial lifetime comorbidity [21, 22]. A meta-analysis of genome-wide association studies of AD in nine clinical samples of European ancestry from seven large, independent studies [23] provided a novel approach to explicitly incorporate comorbidity structure directly into prediction of single nucleotide polymorphisms (SNPs) effects using case–control contrasts and factor score analysis accounting for different ADs in the meta-analysis. This genetic study of AD identified novel genetic variants significantly associated with AD. On chromosome 2p21, three SNPs were detected using a factor score model: rs1067327 within *CAMKMT* (encoding a calmodulin-lysine *N*-methyltransferase), rs1142523 within *SLC3A1* (encoding the large subunit of a heterodimeric dibasic/neutral amino acid transporter), and rs786618 within *PREPL* (encoding a putative prolyl endopeptidase belonging to the prolyl oligopeptidase family). One SNP on chromosome 3q12.3 (rs1709393) within *LOC152225*, (encoding an uncharacterized non-coding RNA locus) was identified using case–control analysis.

In the present study, we first evaluated the findings of Otowa et al. [23] in a large sample of healthy children aged 8–12 years by investigating associations of these SNPs with anxious personality traits in a dimensional approach. In a second step, we analyzed the association of subjective and psychophysiological arousal with the genetic variants found by the meta-analysis of Otowa and colleagues, using a discriminative fear conditioning and generalization paradigm. We expected to find an association of these risk variants with anxiety-relevant traits as well as fear generalization gradients and arousal level. The sample has been recruited with the comprehensive research center “SFB/TRR58—Fear, Anxiety and Anxiety Disorders” funded by the German Research Foundation and, to our knowledge, represents the by far largest sample of children with both data on genetics and fear conditioning/generalization.

## Methods

### Sample

All participants were recruited from primary/secondary schools in the greater region of Wuerzburg, Germany. Inclusion criteria were Caucasian descent (self-report up to third generation), right-handedness (Edinburgh Handedness Inventory; [24]), and fluency in German. Exclusion criteria were manifest or lifetime DSM-IV axis I disorder, severe medical conditions, intake of psychoactive medication, and an IQ < 85 as ascertained by the German version of the Culture Fair Intelligence Test 2 [25]. Absence of DSM-IV axis I disorder was ascertained using the German versions of the Diagnostic Interview for Mental Disorders for Children and Adolescents (Kinder-DIPS; [26]). A total of 475 children between 8 and 12 years of age participated in the study. However, 30 children did not complete the experiment (7 children canceled the experiment prior to acquisition, 6 canceled at acquisition, and additionally 17 children canceled at generalization). Additionally, 42 children were excluded from analysis due to failure of genetic data and/or problems with physiological recordings. Additionally, 56 children were excluded due to genetic relationships with other participants. Thus, a total of 347 children (174 females; mean age: 9.7; SD 1.3) were included in the study. All children gave 2 × 10 ml venous EDTA blood for genetic analyses. Additionally, all participants had to answer to fear-relevant questionnaires (see below): The German version of the Fear Survey Schedule for Children—Revised (PHOKI; [27]), the German version of the Childhood Anxiety Sensitivity Index [(KASI; ([Schneider and Silverman, 2009. Kinder-Angstsensitivitätsindex (KASI). Unpublished Manuskript]), the German version of the Trait scale of the State-Trait Anxiety Inventory for Children (*STAIK-T*; [Unnewehr, Joormann, Schneider, Margraf, 1992. Deutsche Übersetzung des State-Trait Anxiety Inventory for Children. Unpublished Manuskript]) as well as the German version of the Social Phobia and Anxiety Inventory for Children (*SPAIK*; [28]). All participants underwent a discriminant fear conditioning and generalization task. To reach a statistical power of 95%, for the fear conditioning and generalization task (effect size: *f* = 0.025, α err prob = 0.05) a minimal sample size of 24 volunteers and for the psychometric traits (effect size: *d* = 0.85, α err prob = 0.05) a minimum of 31 participants per group are needed (G*Power v3.1.9.2). The study was approved by the ethical committee of the Medical Faculty of the Julius-Maximilian-University of Würzburg (vote 7/08; 106/10) and complied with the latest version of the Declaration of Helsinki. All participants as well as their parents gave written informed consent and each family was paid €50 compensation for their participation.

### Genotyping

For genotyping, four significant SNPs, identified in the study of Otowa et al. [23] (rs1067327, rs786618, rs1142523 and rs1709393—for localization of SNPs, see Suppl Fig. 1), were chosen for analysis. Genotyping data of rs786618, rs1142523 and rs1709393 were generated using MassARRAY system (Agena Bioscience, San Diego; for Primer sequences, see Suppl Table 1) with the iPlex® chemistry following the standard operation procedure and for rs1067327 by restriction analysis of PCR products. PCR fragments were amplified using the primers forward 5′-GCAGGGTAAATTCTTCATTGGT-3′ and reverse 5′-CAGAAAGAGCAATCTCCACAAG-3′ in a 25 µl reaction mix containing 50 ng genomic DNA, 2.5 µl Gold Star buffer, 10 µM of each primer, 2.5 mM of each nucleotide, 25 mM MgCl_2_ and 0.5 U of Taq polymerase. Cycling conditions were 2 min at 94 °C initial denaturation, and 30 s at 94 °C, 30 s at 59 °C, 30 s at 72 °C for 35 cycles and a final extension step at 72 °C for 7 min. PCR products (size 591 bp) were digested with *Csp*I and fragments were visualized on a 2% agarose gel. All four SNPs were polymorphic (minor allele frequency > 0.2%), reached a minimal genotyping call rate of > 96% and did not differ from Hardy–Weinberg equilibrium (*P* > 0.3). Genotypes were determined and analyzed by investigators blinded to phenotypes of participants (sample sizes and allele frequencies are shown in Suppl Table 2).

### Fear-relevant psychometric traits (self-report measures)

The *PHOKI* consists of 98 items on a three-point Likert scale: (0) “never”, (1) “sometimes” and (2) “often”. The *PHOKI* has seven subscales: Fear of Threats and Death (GT), Separation Anxiety (TA), Social Anxiety (SA), Fear of Weirdness (BU), Animal Phobias (TP), Fear of Medical Invasions (ME), and School- and Performance Anxiety (SL). The *KASI* is a self-report scale to measure the level of anxiety, fearfulness and anxiety disorders on 17 items, describing potential reactions to physical symptoms and anxiety on a three-point Likert scale (1) “never”, (2) “sometimes” and (3) “often”, resulting in a sum score between 17 and 51. The *STAIK* is a self-report scale to determine the level of trait anxiety on 20 statements on a three-point Likert scale (1) “almost never”, (2) “sometimes” and (3) “often”, resulting in a sum score between 20 and 60. The *SPAIK* is a self-report scale to determine the level of social anxiety. The *SPAIK* consists of 26 items on a three-point Likert scale (0) “never or almost never”, (1) “sometimes” and (2) “most of the time or ever”, resulting in a sum score between 0 and 52 (descriptive characteristics of fear-relevant psychometric traits of the total sample are given in Suppl Table 3).

### Fear conditioning and generalization task

We used the “screaming lady paradigm” based on Lau et al. [29] and adapted by Schiele, Reinhard et al. [30]. Some studies indicate that the “screaming lady paradigm” represents a more powerful unconditioned stimulus (UCS) than other UCSs employed in research with children and adolescents [30–32].

Pictures of two actresses with neutral facial expression (NimStim Face Stimulus Set; [33]) served as either the CS + or CS − , with one of the two faces being randomly selected as the CS + for each participant. The UCS was a 95-dB female scream (International Affective Digital Sounds system), presented simultaneously with a fearful facial expression of the same actress assigned as the CS + . Four generalization stimuli depicting gradual morphs from CS + to CS − in 20%-steps (GS1–4) were created using the graphics software Sqirlz Morph Version 2.1 (Xiberpix, Solihull, UK). Stimulus presentation was controlled using Presentation software version 17.2 (Neurobehavioral Systems, Inc., Albany, CA, USA). CSs and GSs were presented for 6 s each. The UCS was presented immediately following CS + offset for 1.5 s. Inter-trial intervals varied from 9 to 12 s, during which a white fixation cross was displayed centrally on the screen. Stimulus order was pseudo-randomized so that the same stimulus could not appear more than twice in a row.

The experiment was divided into three consecutive phases: pre-acquisition, acquisition, and generalization. Pre-acquisition consisted of four CS + and four CS − ; no UCS was presented. During acquisition, 12 CS + and 12 CS − were presented. The CS + was paired with the UCS on ten trials. The generalization phase consisted of 12 CS + , 12 CS − , and 12 of each of the four GSs. Half the CS + trials were followed by the UCS to prevent premature extinction. CS- and all GSs were never paired with the UCS. Participants were not informed of the CS–UCS contingencies. Acquisition and generalization trials were separated into two phases, each containing half the trials per phase, i.e., six presentations per stimulus category. Participants were instructed to passively view pictures of two female faces, and that an unpleasant sound would be heard occasionally. They were told that it would be possible to become startled and/or frightened and that participation could be discontinued at any time.

Following (pre)-acquisition and generalization, participants rated each stimulus on arousal and UCS expectancy. Arousals were indicated on nine-point Likert scales, ranging from “very calm” (1) to “very arousing” (9). UCS expectancy was recorded in percent on a scale from 1 to 100 in 10% increments as the probability of an aversive noise following each stimulus. Participants were considered aware of the CS–UCS relationship, if UCS expectancy ratings were higher for the CS + than the CS − . Contingency awareness after acquisition and generalization was determined using the ratings after the second acquisition and generalization phase, respectively.

#### Physiological recordings and data reduction

Throughout the experiments, skin conductance responses (SCRs) were recorded continuously using Brainproducts V-Amp-16 and Vision Recorder software (Brainproducts, Gilching, Germany) at a sampling rate of 1000 Hz and analyzed off-line using Vision Analyzer 2 software (Brainproducts, Gilching, Germany). SCR was recorded from the thenar and hypothenar eminences of the left hand using two Ag/AgCl electrodes. The amplifier delivered a constant current of 0.5 V. The SCR signal was filtered off-line with a high cutoff filter of 1 Hz and a notch filter of 50 Hz. SCR was defined as the base-to-peak difference (in µS) between response onset (900–4000 ms after stimulus onset) and peak (2000–6000 ms after stimulus onset). A minimum response criterion of 0.02 µS was applied, with lower responses scored as 0. SCR data were normalized following an approach described by Dunsmoor et al. [34], i.e., by computing generalization gradients for each phase and block as a function of the response to one stimulus type relative to the sum of responses to all stimuli.

### Statistical analyses

All statistical tests were carried out using SPSS version 24 (SPSS Inc., Chicago, Illinois, USA).

#### Fear-relevant psychometric traits

Descriptive characteristics of genetic variants relative to fear-relevant psychometric traits (PHOKI, KASI, STAIK, and SPAIK) are presented in Suppl Table 4. *T* tests were used to measure differences between the genotypes according to fear-relevant psychometric traits [PHOKI (including seven subscales and a total sum score resulting in 8 PHOKI scales), KASI, STAIK, and SPAIK]. Bonferroni correction was used to correct for multiple testing (significance threshold: *p* < 0.0042, since 12 tests were performed).

#### Fear conditioning and generalization task

Ratings and SCR were analyzed using repeated-measures ANOVAs with the between-subject factor *genotype* (3 groups: rs1067327: CC vs. CG vs. GG; rs786618: CC vs. CT vs. TT; rs1142523: TT vs. CT vs. CC; rs1709393: CC vs. CT vs. TT) and the within-subject factor *stimulus type* (CS + /CS − at acquisition, CS + /CS − /GS1–4 at generalization). Additionally, the within-subject factor *phase* (phase 1 vs. phase 2) was included to detect possible reaction changes between the first and second phase. Due to significant differences in contingency awareness at generalization for rs1709393 (*p* = 0.007), the between-subject factor *awareness* (unaware, aware) was included for analyses relating to rs1709393 at generalization, since awareness of the CS–UCS relationship may influence the conditioned responses [35]. Furthermore, because there were significant sex differences for rs1067327 (*p* = 0.009), rs786618 (*p* = 0.013), and rs1142523 (*p* = 0.022), sex was set as covariate of no interest for analyses concerning these SNPs. ANOVAs were followed by post hoc *t* tests for significant interactions. Alpha was set at 0.05 and Bonferroni correction was applied. Greenhouse–Geisser corrections for non-sphericity were performed where indicated, though uncorrected degrees of freedom are reported for the sake of better readability. Corrected *p* values and partial *η*^2^ for relevant significant results are reported.

## Results

### Fear-relevant psychometric traits

Table 1 shows relevant *p* values of the relations of genotypes and fear-relevant psychometric traits (PHOKI—TP, KASI, STAIKC, and SPAIK; for the sake of clearer arrangement, we focused on PHOKI subscale TP as the most relevant subscale here; results of the other subscales as well as PHOKI total score are presented in Suppl Table 5). For *CAMKMT* rs1067327 and *PREPL* rs786618, CC genotype carriers compared to GG as well as CT and TT genotype carriers, respectively, were significantly associated with lower scores in the fear-relevant psychometric trait PHOKI (TP: *t* (38) =  − 3.21, *p*_rs1067327_ = 0.003; TP: *t* (44) =  − 3.0, *p*_rs786618_ = 0.004; at a nominal level in SL: *p*_rs786618_ = 0.017). No significant associations for STAIKC and/or SPAIK were found (all *p*s ≥ 0.167). For *SLC3A1* rs1142523, at a nominal level CC genotype carriers compared to TT genotype carriers were significantly associated with higher PHOKI scores (TP: *t* (48) = 2.69, *p* = 0.010), conveying genetic risk. Relations with other fear-relevant psychometric traits were not significant (all *p*s ≥ 0.064). *LOC152225* rs1709393 reached no significance at all (all *p*s ≥ 0.046).

**Table 1:** *P* values of relations of fear-relevant psychometric traits and genotypes

	PHOKI (TP) *M* (SD)	*p* value	KASI *M* (SD)	*p* value	STAIK	*p* value	SPAIK	*p* value
*CAMKMT* rs1067327								
CC	2.44 (2.28)	**0.003**	24.67 (3.97)	**0.161**	28.07 (6.25)	**0.281**	8.85 (6.87)	**0.166**
CG	3.99 (3.47)		25.84 (5.83)		29.43 (6.19)		11.23 (8.35)	
GG	4.00 (3.69)		25.87 (5.64)		29.46 (6.50)		10.86 (8.79)	
*PREPL* rs786618								
CC	2.57 (2.39)	**0.004**	24.50 (4.04)	**0.076**	27.90 (6.01)	**0.122**	9.70 (7.34)	**0.356**
CT	4.17 (3.80)		25.97 (5.93)		29.25 (6.20)		10.84 (8.34)	
TT	3.88 (3.58)		25.97 (5.60)		29.90 (6.54)		11.32 (8.99)	
*SLC3A1* rs1142523								
TT	2.67 (2.50)	**0.010**	25.37 (6.00)	**0.545**	28.26 (6.39)	**0.257**	10.70 (7.73)	**0.650**
CT	3.90 (3.50)		25.68 (5.63)		28.98 (6.26)		10.71 (8.44)	
CC	4.18 (3.88)		26.07 (5.69)		29.77 (6.43)		11.15 (8.79)	
*LOC152225* rs1709393								
CC	3.17 (3.10)	**0.072**	24.88 (4.29)	**0.142**	28.21 (5.69)	**0.148**	9.11 (7.49)	**0.123**
CT	4.20 (3.75)		25.90 (5.78)		29.61 (6.82)		11.13 (8.69)	
TT	3.60 (3.21)		25.98 (5.85)		29.55 (5.85)		11.14 (8.30)	

An additive model was used to pertain the *p* values; the above-named *p* values refer to differences between phenotypes that are furthest apart from each other

*M* mean, *SD* standard deviation, *PHOKI* Fear Survey Schedule for Children-Revised (German version), subscale *TP* Animal Phobia, *KASI* Childhood Anxiety Sensitivity Index (German version), *STAIK* Trait Scale of the State-Trait Anxiety Inventory for Children (German version), *SPAIK* Social Phobia and Anxiety Inventory for Children (German version)

*p < .0042; *p*-values that survived Bonferroni correction for multiple testing are highlighted in bold

## Fear conditioning and generalization task

We identified differences in fear learning and generalization according to subjective and psychophysiological arousal in relation to the underlying genetic background, comparing three genotype groups per SNP, respectively, during the acquisition and generalization phases.

### Results during acquisition

Arousal ratings for all relevant SNPs confirmed that the expected conditioning effect as arousal for the CS + was higher than for the CS − (all *ps* ≤ 0.100). For *PREPL* rs786618, the main effect of genotype reached significance (*p* = 0.027), indicating lower arousal in minor allele carriers and higher arousal in major allele carriers, respectively. SCR for *LOC152225* rs1709393 also confirmed the expected conditioning effect as the SCRs to CS + were higher than SCRs to CS − (*p* < 0.001). Additionally, phase reached a significant main effect (*p* < 0.001) with lower SCR in phase 2. Following up a significant Stimulus Type x Phase interaction for *LOC152225* rs1709393 (*F* (1,253) = 6.08, *p* = 0.014, *η*^2^ = 0.02), results showed higher differences between the stimuli in phase 2 indicating better cue discrimination in phase 2.

### Results during generalization

**Fig. 1.**
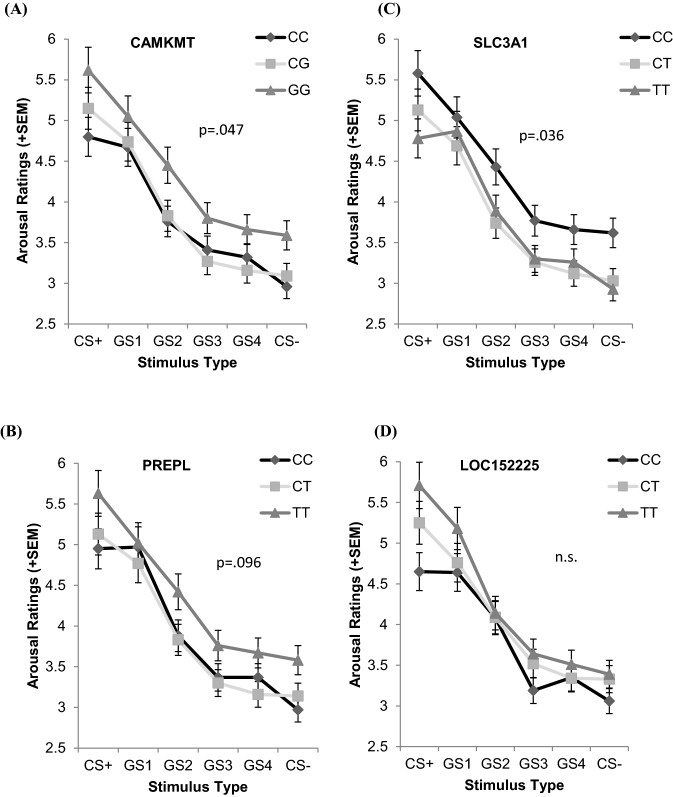
Fear generalization gradients according to arousal ratings for **a** *CAMKMT* rs1067327 genotypes, **b** *PREPL* rs786618 genotypes, **c** *SLC3A1* rs1142523 genotypes, and **d** *LOC152225* rs1709393 genotypes

Significant main effects of stimulus type for all relevant SNPs indicated upward generalization gradients from CS + to CS − according to arousal ratings (all *ps ≤ *0.032) as well as SCR (all *ps* < 0.001). For *CAMKMT* rs1067327 and *SLC3A1* rs1142523 (and marginally for *PREPL* rs786618: *p* = 0.096), significant main effects of genotypes were found in arousal ratings (*CAMKMT* rs1067327: *F* (2,336) = 3.08, *p* = 0.047, *η*^2^ = 0.02; *SLC3A1* rs1142523: *F* (2,337) = 3.34, *p* = 0.036, *η*^2^ = 0.02). For *CAMKMT* rs1067327, higher arousal in GG genotype carriers (Fig. 1a) (and in TT genotype carriers for *PREPL* rs786618, respectively; Fig. 1b) was found, and higher arousal in CC genotype carriers was found for *SLC3A1* rs1142523 (Fig. 1c). Additionally, for *PREPL* rs786618, a significant Genotype x Phase interaction effect was found for SCR (*F* (2,246) = 3.83, *p* = 0.023, *η*^2^ = 0.02). Post hoc tests, however, reached no significance. No significant main effect was found for genotypes related to *LOC152225* rs1709393 (Fig. 1).

For arousal ratings, contingency awareness played a significant role within the analyses with a Phase × Genotype × Awareness interaction (*F*(2,328) = 3.25, *p* = 0.040, *η*^2^ = 0.02), a significant main effect of awareness, and a significant Stimulus Type × Awareness interaction resulting in a three-way interaction of Stimulus Type × Phase × Awareness for *LOC152225* rs1709393 (*F*(5,1516) = 8.70, *p* < 0.001, *η*^2^ = 0.03). Post hoc tests indicated that aware participants were better at discriminating stimuli compared to unaware participants, and this was true for phase 1 and phase 2, but with better stimuli discrimination of aware participants in phase 2, and with TT genotype carriers being more often aware than CC genotype carriers. Furthermore, awareness played also a role in Arousal ratings as indicated by a significant main effect of awareness for *LOC152225* rs1709393 (*F*(1,328) = 8.44, *p* = 0.004, *η*^2^ = 0.02) with higher SCR in aware participants.

## Discussion

The present study investigated the impact of four genetic risk variants, recently identified as associated with pathological anxiety in adults in a meta-analysis of genome-wide association studies of anxiety disorders (AD) [23], on fear- and anxiety-relevant personality traits in a large sample of typically developing children aged 8 to 12 years. In this study, we aimed to extend the findings of Otowa et al. [23] by applying a dimensional approach and including physiological conditioning/generalization data. The chosen age range marks a developmental period, in which a wide range of AD manifest for the first time. Assuming that common variants play a crucial role in AD susceptibility, we additionally analyzed those risk variants with genome-wide significance [23] in healthy children with respect to generalization gradients according to subjective and objective measures of arousal, since overgeneralization and heightened arousal are assumed risk factors in the pathogenesis of AD [12, 14, 15, 36].

First, we evaluated the findings by Otowa et al. [23] in a sample of healthy 8–12 years old children in a dimensional approach, indicating correlations of fear- and anxiety-relevant personality traits and heightened anxiety sensitivity with the analyzed risk genotypes. In more detail, analyses of the used fear- and anxiety-relevant dimensional personality traits showed that relating to *CAMKMT* rs1067327, *PREPL* rs786618, and at a nominal level *SLC3A1* rs1142523, the minor allele was associated with lower scores in PHOKI (basically TP) and, thus, the minor allele functioned more in a protective manner. The other dimensional questionnaires yielded similar effects, but reached no significance, presumably due to lack of power and/or little variance. We could only establish the link in chromosome 2, but reached no significance for chromosome 3, again possibly due to small sample sizes and due to the little diverse dimensional phenotypes. On the other hand, the SNP in chromosome 3 may not be associated with alterations in arousal, but with other RDoC, such as for example valence. Thus, further exploration is necessary. The reason for the sole genetic association with the PHOKI scores might possibly be due to the age of the participants. Psychopathological differentiation between categorical disorders in children is often hampered by the child’s difficulty differentiating distinct constructs and expressing themselves. Thus, while KASI and STAIK measure an anxiety-prone personality and SPAIK scores assess social anxious traits, the PHOKI relates to specific and clear-cut fears that may be readily distinguishable for children.

Further, we found that the analyzed risk genotypes on chromosome 2 are associated with altered arousal to threat/safety stimuli during fear learning, but not to enhanced generalization per se. In detail, while for the overall sample conditioning was successful and the expected upward generalization gradients from CS + to CS − could be identified, there were main effects of genotypes for *CAMKMT* rs1067327 and *SLC3A1* rs1142523, but no Genotype × Stimulus Type interaction effects. The homozygote genotypes with major allele frequency (GG in *CAMKMT* rs1067327, TT in *PREPL* rs786618, CC in *SLC3A1* rs1142523) were consistently associated with higher arousal ratings. However, results according to SCR yielded no significant effects, possibly due to larger physiological variance in children and the high error rate for physiological measures of arousal, although similar trends were found. Our results are in line with previous studies showing that anxious participants rated emotional images as more arousing and reported more fear than healthy participants, and that there were inconsistencies between self-reports and physiological states (12, 37, 38]. However, our results must be interpreted carefully, since even though the here presented sample size represents the largest child sample to date with combined genetic and fear conditioning/generalization data, it is still comparatively small for investigating genetic effects. Thus, results must be analyzed and reviewed in larger samples, integrating healthy as well as pathologically anxious participants.

Moreover, consistent with previous results of our research group [30], participants, who were aware of the contingency, were better at stimuli discrimination and this was especially true for phase 2. Additionally, aware participants showed generally higher SCR than unaware participants and TT genotype carriers were more often aware than CC genotype carriers according to *LOC152225* rs1709393. This finding could be explained by attentional processes, meaning that TT genotype carriers showed more attention toward threat. This would match a study showing that clinically anxious participants consistently shifted attention toward threat, resulting in reduced detection latencies for probes appearing in the vicinity of such stimuli, supporting the existence of anxiety-related encoding bias that may contribute to the maintenance of such mood disorders [37]. However, this finding must be verified in larger samples with and without AD.

The results expand results of the named meta-analysis of genome-wide association studies of AD, but require replication and further exploration in other samples. Importantly, the effect found in our data regarding rs1067327 was contrarious to what is specified in the meta-analysis of Otowa et al. [23]. Whereas in our sample, anxiety traits and increased arousal were positively associated with the major G allele, the results of Otowa et al. [23] indicated a positive association between the C allele and the corresponding anxiety phenotype. This could be due, for instance, to different samples. Whereas in the named meta-analysis adults were included, we exclusively investigated children. Additionally, children of our study were healthy, whereas participants with different manifest anxiety disorders were included in the named meta-analyses. Furthermore, a reduction of variation could have occurred due to more anxious children cancelling the experiment and thus having reduced our liability scale on the upper anxiety end. Indeed, the 30 children that did cancel were significantly more anxious relating to STAIK scores than the other participants, but relating to other self-report measures and also to genotype there were no statistical differences between groups. Unfortunately, we were unable to determine the directions of the associations between rs786618 as well as rs1142523 and the corresponding anxiety phenotype as described in Otowa et al. [23].

How could the three SNPs from the chromosome 2p21 region be mechanistically linked to the development of increased anxiety sensitivity and higher arousal states during fear learning? All three SNPs are located in a very gene-rich region spanning only approximately 83 kb (see Suppl Fig. 1) and are therefore not inherited independently, thus not allowing to attribute the association to a specific gene. The rs1142523 and rs1067327 SNPs are located in intronic regions in their respective genes (*SLC3A1* and *CAMKMT*), whereas rs786618 is located in the 5′UTR of *PREPL*. It is not known if any of these SNPs influence the splicing pattern, expression levels, or transcript stability of their respective genes. Interestingly, a yet uncharacterized microRNA gene is located intronic in *CAMKMT* as well. According to databases, all these transcripts can be detected in brain tissue. In humans a deletion of the 2p21 region including four genes (*PP2Cβ*, *SLC3A1*, *PREPL* and *CAMKMT*) was described as a syndrome (MIM #606,407) associated with cystinuria, intellectual disability, mitochondrial disease, hypotonia and facial dysmorphism [38]. Mice deficient for *CAMKMT* expression show developmental delay, mitochondrial defects, and brain alterations [39]. Motor learning and complex coordination and learning of aversive stimuli are also impaired, pointing to the possibility that *CAMKMT* could be a reasonable candidate for further experiments. Knockout mice for *PREPL* suffer neonatal hypotonia and diminished growth [40] making this gene a less likely candidate, similarly to *SLC3A1* null mice displaying cystinuria [41]. Further experiments are needed to investigate how these human SNPs might be associated with anxiety and arousal and what biological mechanisms are associated with this phenotype.

Taken together, our results expand findings by Otowa et al. [23] evaluating the findings on adult patient samples in healthy children aged 8–12 years and showing that the anxiety disorder risk SNPs are associated both with (1) dimensional anxiety traits and (2) arousal. As this is the first study evaluating the findings by Otowa et al. [23] in a dimensional approach in a children sample, our results must be replicated in larger samples and with other robust approaches. These should also include additional genome-wide significant SNPs generated in a very recent study investigating more than 150.000 individuals in the UK [42], or by including SNPs identified in a recent twin study in generalized anxiety disorder [43]. Heightened anxiety traits and arousal could serve as trans-diagnostic frameworks in terms of the RDoC approach and accelerate the definition of the genetic underpinnings of anxiety disorders.

## Electronic supplementary material

Below is the link to the electronic supplementary material.

Supplementary file1 (DOCX 83 kb)
