# Supplementary material for: Anxiety risk SNPs on chromosome 2 modulate arousal in children in a fear generalization paradigm
Source: Eur Child Adolesc Psychiatry. 2019 Dec 21;29(9):1301–10. doi: 10.1007/s00787-019-01458-7 (PMC7497385; doi:10.1007/s00787-019-01458-7)
Supplement: Supplementary file 1 — Supplementary file1 (DOCX 83 kb) [file 787_2019_1458_MOESM1_ESM.docx]

Supplementary Material

**Table 1.** **Primer sequences in 5’-3’ direction used for Sequenom’s MassArray® system.**

| **SNP ID** | **Primary PCR Primer 1** | **Primary PCR Primer 2** | **Extend Primer** |
| --- | --- | --- | --- |
| rs786618 | ACGTTGGATGAGTCTCACTCCATCATCCAG | ACGTTGGATGTTTGAACCCAGGACATGGAG | GAAGTGCAGCAGTGTGATCT |
| rs1142523 | ACGTTGGATGGCCTGGCCTGCAAAATTTGT | ACGTTGGATGGGGCCCATAGTTGATAGAAC | AGACACAAGAAATGATTTAAATAT |
| rs1709393 | ACGTTGGATGCGCTTAGGATTTGCACACTG | ACGTTGGATGAGCACGAAACCATGCTAGTT | GATTTGCACACTGTATCTTTT |

**Table 2. Frequency of the genetic variants**

| SNP | Chr | A12 | N (%) | N (%) | N (%) | N (%) | Frq_mean (Meta-analysis)_ | Frq  1000G | Frq TOPMED | Frq GnomAD | Frq ALSPAC |
| --- | --- | --- | --- | --- | --- | --- | --- | --- | --- | --- | --- |
| CAMKMT rs1067327 | **2** | **CG** | **00**  7 (1.7) | **CC**  32 (7.9) | **CG**  175 (43.4) | **GG**  189 (46.9) | **0.38** (0.34) | **0.48** | **0.44** | **0.44** | **0.32** |
| *PREPL*  rs786618 | **2** | **CT** | **00**  12 (3.0) | **CC**  35 (8.7) | **CT**  178 (44.2) | **TT**  178 (44.2) | **0.41** | **0.43** | **0.49** | **0.49** | **0.33** |
| *SLC3A1*  rs1142523 | **2** | **TC** | **00**  6 (1.5) | **TT**  32 (7.9) | **CT**  160 (39.7) | **CC**  205 (50.9) | **0.36** | **0.41** | **0.35** | **0.36** | **0.31** |
| *LOC152225* rs1709393 | **3** | **TC** | **00**  13 (3.2) | **TT**  144 (35.7) | **CT**  190 (47.1) | **CC**  56 (13.9) | **0.53** (0.58) | **0.48** | **0.46** | **0.46** | **0.42** |

SNP: single nucleotide polymorphism; Chr: chromosome; Frq: frequency of allele1 (compared to results of the meta-analysis of GWAS of anxiety disorders mentioned in Otowa et al., 2016 and compared to different high-throughput frequency analysis taken from the SNP reference report at (https://www.ncbi.nlm.nih.gov/snp/rs...)).

**Table 3**. **Descriptive characteristics of fear-relevant psychometric traits of the total sample**

|  | **M** | **SD** |
| --- | --- | --- |
| **PHOKI** | 47.12 | 30.83 |
| **KASI** | 25.84 | 5.68 |
| **STAIK** | 29.38 | 6.34 |
| **SPAIK** | 10.90 | 8.54 |

*M* = mean; *SD* = standard deviation; *PHOKI* = Fear Survey Schedule for Children – Revised (German version); *KASI* = Childhood Anxiety Sensitivity Index (German version); *STAIK* = Trait scale of the State-Trait Anxiety Inventory for Children (German version); *SPAIK* = Social Phobia and Anxiety Inventory for Children (German version).

**Table 4**. **Descriptive characteristics of the genetic variants relative to fear-relevant psychometric traits**

|  | **CAMKMT rs1067327**  **00** | **CC** | **CG** | **GG** |  |
| --- | --- | --- | --- | --- | --- |
| **PHOKI M(SD)**  **KASI M (SD)**  **STAIK M (SD)**  **SPAIK M (SD)** | 60.71 (51.09)  29.86 (7.94)  31.57 (7.96)  12.71 (12.46) | 40.11 (24.73)  24.67 (3.97)  28.07 (6.25)  8.85 (6.87) | 47.21 (32.31)  25.84 (5.83)  29.43 (6.19)  11.23 (8.35) | 47.60 (29.35)  25.87 (5.64)  29.46 (6.50)  10.86 (8.79) | |
|  | ***PREPL*** **rs786618**  **00** | **CC** | **CT** | **TT** | |
| **PHOKI M(SD)**  **KASI M (SD)**  **STAIK M (SD)**  **SPAIK M (SD)** | 55.0 (35.52)  26.00 (6.94)  28.08 (7.18)  9.09 (8.50) | 38.73 (22.90)  24.50 (4.04)  27.90 (6.01)  9.70 (7.34) | 47.02 (33.17)  25.97 (5.93)  29.25 (6.20)  10.84 (8.34) | 48.30 (29.37)  25.97 (5.60)  29.90 (6.54)  11.32 (8.99) | |
|  | ***SLC3A1* rs1142523**  **00** | **CC** | **CT** | **TT** | |
| **PHOKI M(SD)**  **KASI M (SD)**  **STAIK M (SD)**  **SPAIK M (SD)** | 46.67 (28.54)  25.17 (5.91)  32.17 (7.08)  8.50 (8.55) | 49.11 (30.06)  26.07 (5.69)  29.77 (6.43)  11.15 (8.79) | 45.73 (32.97)  25.68 (5.63)  28.98 (6.26)  10.71 (8.44) | 41.22 (24.87)  25.37 (6.00)  28.25 (6.39)  10.70 (7.73) | |
|  | ***LOC152225* rs1709393**  **00** | **CC** | **CT** | **TT** | |
| **PHOKI M(SD)**  **KASI M (SD)**  **STAIK M (SD)**  **SPAIK M (SD)** | 67.75 (45.79)  27.00 (7.49)  29.69 (7.83)  12.67 (12.87) | 42.23 (29.52)  25.02 (4.28)  28.12 (5.66)  9.02 (7.26) | 47.90 (30.31)  25.90 (5.78)  29.61 (6.82)  11.13 (8.69) | 45.94 (29.78)  25.98 (5.85)  29.55 (5.85)  11.14 (8.30) | |

*M* = mean; *SD* = standard deviation; *PHOKI* = Fear Survey Schedule for Children – Revised (German version); *KASI* = Childhood Anxiety Sensitivity Index (German version); *STAIK* = Trait scale of the State-Trait Anxiety Inventory for Children (German version); *SPAIK* = Social Phobia and Anxiety Inventory for Children (German version).

**Table 5. P-values of relations between PHOKI and genotypes**

|  |  | **PHOKI total**  ***M (SD)*** | **p-value** | **GT**  ***M(SD)*** | **p-value** | **TA**  ***M(SD)*** | **p-value** | **SA**  ***M(SD)*** | **p-value** | **BU**  ***M(SD)*** | **p-value** | **ME**  ***M(SD)*** | **p-value** | **SL**  ***M(SD)*** | **p-value** |
| --- | --- | --- | --- | --- | --- | --- | --- | --- | --- | --- | --- | --- | --- | --- | --- |
| ***CAMKMT* rs1067327** | **CC** | 40.11 (24.73) | .211 | 11.41 (7.42) | .369 | 10.04 (5.35) | .437 | 3.78 (3.67) | .441 | 6.89 (6.56) | .141 | 2.67 (2.40) | .180 | 2.81 (2.65) | .172 |
|  | **CG** | 47.21 (32.31) |  | 11.54 (7.87) |  | 10.70 (7.13) |  | 4.43 (4.12) |  | 9.41 (8.47) |  | 3.48 (3.14) |  | 3.72 (3.54) |  |
|  | **GG** | 47.60 (29.35) |  | 12.34 (7.56) |  | 11.06 (6.47) |  | 4.25 (3.67) |  | 8.76 (7.38) |  | 3.48 (2.99) |  | 3.75 (3.78) |  |
| ***PREPL* rs786618** | **CC** | 38.73 (22.90) | .094 | 11.03 (7.30) | .299 | 9.47 (4.95) | .137 | 3.43 (3.04) | .162 | 7.03 (6.41) | .152 | 2.63 (2.28) | .115 | 2.57 (2.40) | .020 |
|  | **CT** | 47.02 (33.17) |  | 11.52 (7.89) |  | 10.55 (7.19) |  | 4.29 (4.05) |  | 9.42 (8.61) |  | 3.40 (3.13) |  | 3.73 (3.59) |  |
|  | **TT** | 48.30 (29.37) |  | 12.45 (7.61) |  | 11.34 (6.48) |  | 4.48 (3.85) |  | 8.83 (7.40) |  | 3.55 (3.01) |  | 3.81 (3.37) |  |
| ***SLC3A1* rs1142523** | **TT** | 41.22 (30.06) | .197 | 11.78 (6.79) | .186 | 9.74 (5.16) | .195 | 3.48 (3.04) | .178 | 7.93 (6.91) | .479 | 2.93 (2.57) | 297 | 2.70 (2.52) | .064 |
|  | **CT** | 45.73 (32.97) |  | 11.30 (8.09) |  | 10.36 (7.26) |  | 4.19 (4.03) |  | 9.16 (8.45) |  | 3.39 (3.18) |  | 3.50 (3.51) |  |
|  | **CC** | 49.11 (30.06) |  | 12.49 (7.53) |  | 11.38 (6.52) |  | 4.55 (3.92) |  | 8.97 (7.65) |  | 3.57 (3.02) |  | 4.00 (3.48) |  |
| ***LOC152225***  **rs1709393** | **CC** | 42.23 (29.52) | .253 | 11.15 (8.19) | .340 | 9.81 (6.32) | .263 | 4.00 (3.66) | .488 | 7.69 (7.55) | .200 | 2.92 (2.52) | .193 | 3.48 (3.56) | .541 |
|  | **CT** | 47.90 (30.31) |  | 12.31 (7.76) |  | 11.05 (6.81) |  | 4.45 (4.07) |  | 8.64 (7.40) |  | 3.55 (3.05) |  | 3.75 (3.20) |  |
|  | **TT** | 45.94 (29.78) |  | 11.44 (7.33) |  | 10.63 (6.50) |  | 4.13 (3.64) |  | 9.40 (7.88) |  | 3.30 (3.02) |  | 3.51 (3.54) |  |

*M* = mean; *SD* = standard deviation; PHOKI total = Fear Survey Schedule for Children – Revised (German version), subscale GT = Fear of Threats and Death; TA = Separation Anxiety; SA = Social Anxiety; BU = Fear of Weirdness; ME = Fear of Medical Invasions; SL = School- and Performance Anxiety; an additive model was used to pertain the p-values; the above-named p-values refer to differences between phenotypes that are furthest apart from each other.

**
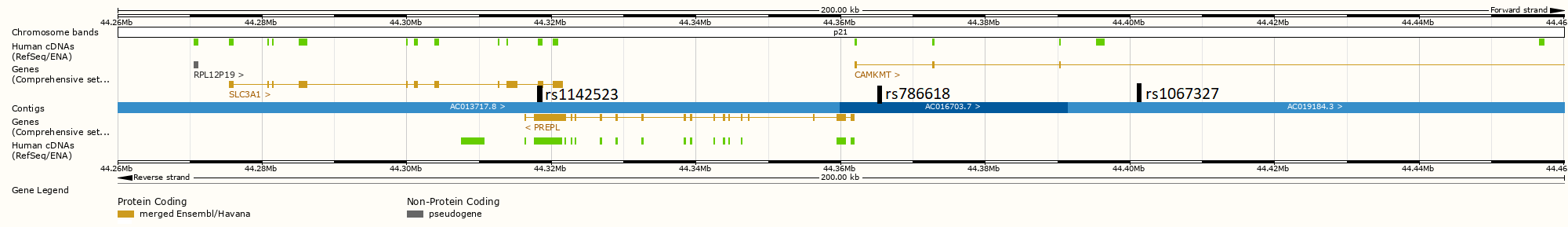
**

**Fig 1.** Localization of the analysed SNPs on chromosome 2.

Figure was generated with the ensemble genome browser according to the latest genome build (Ensembl release 92, April 2018) GRCh38.p12 (GCA_000001405.27).
